# IgG Autoantibody to Brain Beta Tubulin III Associated with Cytokine Cluster-II Discriminate Cerebral Malaria in Central India

**DOI:** 10.1371/journal.pone.0008245

**Published:** 2009-12-14

**Authors:** Devendra Bansal, Fabien Herbert, Pharath Lim, Prakash Deshpande, Christophe Bécavin, Vincent Guiyedi, Ilaria de Maria, Jean Claude Rousselle, Abdelkader Namane, Rajendra Jain, Pierre-André Cazenave, Gyan Chandra Mishra, Cristiano Ferlini, Constantin Fesel, Arndt Benecke, Sylviane Pied

**Affiliations:** 1 Equipe PIME CNRS, Inserm U547, Institut Pasteur de Lille, Pôle Universitaire Nord, France; 2 National Centre for Cell Science, Pune, Pune (Maharashtra), India; 3 Institut de Recherche Interdisciplinaire CNRS USR3078 Univ. Lille I, II, and Institut des Hautes Études Scientifiques, Bures sur Yvettes, France; 4 Laboratory of Antineoplastic Pharmacology, Università Cattolica Sacro Cuore, Rome, Italy; 5 Institut Pasteur, Plate-Forme de Protéomique, CNRS URA 2185, Paris, France; 6 K.T.S. Hospital, Gondia District, Maharashtra, India; 7 Université Pierre et Marie Curie–CNRS U7087, and Institut Pasteur, Paris, France; 8 Instituto Gulbenkian de Ciência, Oeiras, Portugal; Federal University of São Paulo, Brazil

## Abstract

**Background:**

The main processes in the pathogenesis of cerebral malaria caused by *Plasmodium falciparum* involved sequestration of parasitized red blood cells and immunopathological responses. Among immune factors, IgG autoantibodies to brain antigens are increased in *P. falciparum* infected patients and correlate with disease severity in African children. Nevertheless, their role in the pathophysiology of cerebral malaria (CM) is not fully defined. We extended our analysis to an Indian population with genetic backgrounds and endemic and environmental status different from Africa to determine if these autoantibodies could be either a biomarker or a risk factor of developing CM.

**Methods/Principal Findings:**

We investigated the significance of these self-reactive antibodies in clinically well-defined groups of *P. falciparum* infected patients manifesting mild malaria (MM), severe non-cerebral malaria (SM), or cerebral malaria (CM) and in control subjects from Gondia, a malaria epidemic site in central India using quantitative immunoprinting and multivariate statistical analyses. A two-fold complete-linkage hierarchical clustering allows classifying the different patient groups and to distinguish the CM from the others on the basis of their profile of IgG reactivity to brain proteins defined by PANAMA Blot. We identified beta tubulin III (TBB3) as a novel discriminant brain antigen in the prevalence of CM. In addition, circulating IgG from CM patients highly react with recombinant TBB3. Overall, correspondence analyses based on singular value decomposition show a strong correlation between IgG anti-TBB3 and elevated concentration of cluster-II cytokine (IFNγ, IL1β, TNFα, TGFβ) previously demonstrated to be a predictor of CM in the same population.

**Conclusions/Significance:**

Collectively, these findings validate the relationship between antibody response to brain induced by *P. falciparum* infection and plasma cytokine patterns with clinical outcome of malaria. They also provide significant insight into the immune mechanisms associated to CM by the identification of TBB3 as a new disease-specific marker and potential therapeutic target.

## Introduction

Malaria remains a major cause of morbidity and mortality in humans, resulting 350–500 million clinical cases and over one million deaths annually [1]. *Plasmodium falciparum* infection generates pleiomorphic clinical outcomes, from asymptomatic to severe syndromes depending on transmission intensity, age of the individuals and on the immunity and the genetic background of the populations [2], [3], [4]. Anemia and cerebral malaria (CM) are the most severe manifestations and deaths occur by CM in children and young adults in area of high transmission [5]. CM is characterized by a range of acute neurological manifestations including a diffuse encephalopathy, alteration in levels of consciousness, deep coma and seizure preceding death [6], [7]. Sequestration of parasitized erythrocytes in cerebral blood vessels is often associated to CM [8]. Adhesion of blood stage parasite has been considered to lead to a decrease of the blood flow and to contribute to the induction of brain damage and coma during CM [9], [10]. Additionally, CM is also considered to be the result of an immunopathological process involving both lymphocytes and proinflammatory (Th1) cytokines such as TNFα, levels of which are increased in affected patients [11]–[13]. Thus, the outcome of *P. falciparum* infection may depend on a fine balance between appropriate and inappropriate immune responses [14], [15]. Although the occurrence of numerous metabolic, pathological and physiological abnormalities has been demonstrated during CM, the mechanisms leading to progression into complicated disease have not been yet adequately explained. Particularly, pathogenic roles for autoantibodies are not defined in CM.

When exposed to *Plasmodium* parasite, the host immune response is characterized by a polyclonal B-cell activation and a hyper gamma-globulinemia [16], [17]. Among antibodies produced some of them recognize autoantigens [17], [18]. High levels of antibodies against phospholipids, cardiolipin, ssDNA, dsDNA, and rheumatoid factors are correlated with disease severity in *P. falciparum*-infected patients [19]–[22]. However, their role in pathophysiology of CM remains unclear. Recently, by studying several cohorts of children manifesting different disease spectrums induced by *P. falciparum* from a hyper endemic area of Gabon, we demonstrated that antibody mediated self-reactive response may contribute to the pathogenesis of CM. Thus, in these children we observed a significant increase of the repertoire of plasmatic IgG reacting with human brain antigens with disease severity [23]. Interestingly, CM patients developed a high IgG autoantibody response to brain α II spectrin which is significantly associated with increased plasma concentrations of TNFα [23]. These autoantibodies may or may not cause damage. The relationship between CM and antibody dependent auto-immune reactions has been also illustrated by the occurrence of autoantibodies against voltage-gated calcium channels in African populations [24]. Multiple mechanisms underlie the production of autoantibodies such as a polyclonal activation of B cells due to stimulation by parasitic mitogens [25], a stimulation of specific B cells by molecular mimetism [26], [27], or even a deregulation of the B cells function [25], [28]. Other mechanisms such as apoptosis of brain endothelial cells occurring during cerebral malaria could also be source of release of the auto antigens [29], [30].

In this study, we extended our analysis to an Indian population with genetic backgrounds, endemic and environmental status different from the Gabonese population to determine if autoreactive antibodies specific to brain antigens are present in CM patients and could play a role in malaria pathogenesis. We used a multidisciplinary approach based on quantitative immunoprinting associated to biostatistics to study the autoantibody repertoire to brain antigens in several groups of *P. falciparum* infected patients from an epidemic area of central India manifesting different clinical spectra of the disease. We found that the different clinical malaria phenotypes can be discriminate according to their profile of IgG reactivity to brain antigens. Furthermore, we identified a novel discriminant brain antigen, the beta tubulin III (TBB3), targeted by circulating IgG in the prevalence of CM. TBB3, a cytoskeleton protein, is mainly expressed in neural tissue [31]. Finally, we show that IgG reactivity to TBB3 is strongly correlated with elevated levels of the previously described cytokine cluster II, composed of IL10, TNFα, TGFβ and IL1β, that characterized CM in the same group of patients [32].

## Materials and Methods

### Ethics statement

This study was conducted according to the principles expressed in the Declaration of Helsinki. The study was approved by the Institutional Review Board of NCCS, Institut Pasteur Paris and Gondia hospitals. The study design was also approved by the National health office ethics committee in India. All patients or relatives provided written informed consent for the collection of samples and subsequent analysis.

### Study area and subjects

Blood samples were collected from the individuals living in villages in and around Gondia town, an epidemic region in central India. Gondia is a low transmission region, and know as endemic area for the last 20 years. *P. falciparum* appeared in Gondia over the last 10 years [33]. The subjects were divided into the following groups. Group 1 consisted of subjects, who had CM within the past 6 months and recovered (ex-CM), healthy malaria endemic controls (EC) were patient's relatives (brothers/sisters/parents) who accompanied the patient to the hospital and not had malaria for at least the preceding 2 years, nor were they clinical asymptomatic carriers; and malaria non-endemic controls (NEC) were the subjects residing in the Pune city with no history of malarial disease for ≥5 years. Group 2 consisted of patients infected with *P. falciparum* having different clinical status, according to the criteria defined by the World Health Organization [34] i.e. mild malaria (MM), severe non-cerebral malaria (SM) and cerebral malaria (CM). Samples from infected groups were collected during the period of high malaria transmission. Patients with MM (hemoglobin ≥8 g/dl, parasitaemia asexual ≥10000/µl, fully consciousness) were not hospitalized and SM patients were also complete conscious and displayed good verbal response to the doctor's questions. Patients with CM were at coma stages I and III. Patients with severe malaria (SM or CM) received intravenous quinine (25 mg/kg/day) with 5% or 10% glucose solution for non-hypoglycaemic or hypoglycaemic patients for five days. The patients with severe anaemia underwent blood transfusion. Most of the CM patients recovered from disease in one or two weeks and have been discharged from the hospital. The clinical history and informed written consent were obtained from all the subjects and a demographic profile was recorded.

A Pool of CM serum was constituted with samples from patients showing a high reactivity to brain antigens. An EC pool of 5 sera was randomly chosen as negative control.

### Blood samples collection and parasite assessment

Ten milliliters of whole blood was collected from each subject by vein puncture in sterile EDTA tubes or in sterile vacutainers during 2001–2003 from different hospitals in and around Gondia town. Plasma was obtained by centrifuging the blood samples at 4500 *g* for 15 min and stored at −80°C until further use. Parasitemia was assessed, on thin blood smear, by counting asexual forms of *P. falciparum* under a light microscope after Giemsa staining. The total numbers of infected and uninfected erythrocytes from 10 fields (magnification, X100) were counted, and parasitemia were calculated.

### Extraction of antigen

#### Parasites extract

Parasite antigen prepared from synchronous cultures of a field derived *P. falciparum* parasite line *FAN5HS* [33], ≥25% parasitemia, was used. The parasitized red blood cells (pRBC) were washed five times in sterile PBS and then lysed by lysis buffer containing protease inhibitors and briefly sonicated. The contents of the tube were agitated by cyclo-mixing and then centrifuged at 6,000 rpm for 30 min at 4°C. The supernatant was collected in a separate tube and the pellet was discarded. Aliquots of the antigen were frozen at −70°C until use. Parasite protein was quantified by the standard Bradford method [35]. The concentration of the parasite line *FAN5HS* was 1.2 mg/ml.

#### Normal RBC extracts

Normal red blood cell (RBC) extract was prepared from the same batch of RBCs used for culturing the parasites [33] and followed the same procedure as previously described for pRBCs.

#### Human brain extract

The protein extraction of brain was done by homogenization of whole brain taken from a healthy Cuban national, who died accidentally and never had malaria [36], [37]. The brain tissue was suspended in extraction buffer containing 60 mM Tris, 2% SDS, 100 mM Dithiothreitol (DTT) and protease inhibitors: 1 µg/ml Aprotinine, 1 µg/ml Pepstatine, 50 µg/ml n-α-todyl-L-lysine chloromethyl ketone (TLCK). After centrifugations at 10000 rpm at 4°C for 10 minutes, the supernatant was transferred into a clean tube and protein contents were estimated using a commercial available kit (BCATM protein assay kit, Pierce, France). The concentration of the brain extract was 3 mg/ml. Commercially available brain extract (Protein MEDLEY, Ozyme, France) was also used to compare auto reactivity to an external standard extract in the same samples.

### Determination of IgG and IgM levels

#### Total IgG and IgM

The total IgG and IgM were quantified by “Sandwich ELISA” [23]. Briefly, 96 flat-bottomed plates were coated with monoclonal antibodies directed against human IgG or IgM (5 µg/ml) and left for adsorption at 4°C overnight. Plates were washed 5 times with PBS-0.1% Tween 20 and blocked with PBS-1% Gelatin at 37°C for 1 hour. Wells were incubated with serum samples diluted at 1∶100 in PBS-1% Gelatin-0.1% Tween 20 for 1 h at 37°C. Excess antibody was removed by 5 PBS-0.1% Tween 20 washings and then plates were incubated with peroxydase-labeled anti-human IgG and anti-human IgM (1∶2000 in PBS-1% Gelatin, 0.1% Tween 20) at 37°C for 1 h. The assay was developed by adding the enzyme substrate (O-phenylenediamine diluted to 0.3 mg/ml in Phosphate-citrate buffer in the presence of hydrogen peroxide). After appearance of yellow color in negative wells, the reaction was stopped with 10% SDS. The OD was measured at 450 nm using an Emax ELISA plate reader and results were analysed by the Sofmax software.

### Specific anti-parasite and anti-brain IgG and IgM

The anti *P. falciparum* and anti-brain IgG and IgM were analyzed by direct ELISA. Flat-bottomed 96 well plates were coated overnight at 4°C with 5 µg/ml parasite line (*FAN5HS*) or brain antigen. After washing, the plates were saturated with PBS-1% Gelatin for 1 hour at 37°C. Subsequently the sera were diluted (anti-parasite and anti-brain IgG 1/1000 and 1/500 respectively and IgM 1/500 and 1/500 respectively) in PBS-1% Gelatin, 0.1% Tween 20 and added in duplicate to the wells and incubated at 37°C for 1 hour. The plates were washed five times in PBS 0-1% Tween 20 and incubated for 1 hour at 37°C following the addition of peroxydase - conjugated human anti-IgG or anti-IgM (1∶4000 and 1∶2000 for anti-parasite and anti-brain respectively in PBS-1% Gelatin, 0.1% Tween 20). The process of revelation is the same as the total IgG and IgM.

### Cytokine quantification

The levels of cytokines (IL1β, IL2, IL4, IL6, IL10, IL12, TGFβ, TNFα and IFNγ) in plasma were estimated by use of Opti-EIA kits (BD-Pharmingen); the results of which are already published earlier [32].

### Immunobloting using PANAMA–blot method

Patterns of recognition of brain proteins by plasma IgG were detected by quantitative immunoblotting as described earlier [23], using a protein extract from the brain of a healthy individual as the source of antigens and normal RBC as control as described above. Briefly, normal human brain and RBC protein extracts (300 µg protein/gel) were separated by a standard SDS-PAGE in a 10% polyacrylamide gel. The proteins were transferred onto nitrocellulose membranes (Schleicher & Schuell, Dassel, Germany) by semi-dry electro transfer method (Pasteur Institute, Paris, France). Membranes were then incubated with patient plasma samples diluted 1∶20 in PBS-0.1% Tween 20 (non-adjusted assay) in a Cassette Miniblot System (Immunetics, Cambridge, MA, USA). The immunoglobulin reactivities were detected by incubation with γ chain-specific secondary rabbit anti-human IgG coupled to alkaline phosphatase (Sigma-Aldrich, France). Revelation was done by using BCIP/NBT. As described [36] dried membranes were then scanned by a high resolution scanner (600 DPI) using an 8-bit linear grayscale. Subsequently, transferred proteins on the membranes were stained with colloidal gold (Protogold, BritishBioCell, Cardiff, GB), and the stained membranes scanned again. Using colloidal gold staining, immunoreactivity profiles were adjusted for migration inequalities, so that equivalent immunoreactivities could be rescaled to equivalent positions on a common standard migration scale within and between membranes. Intensities were adjusted between membranes by a standard, consisting of a pool of serum from Gabonese CM patients [23] that was replicated twice on each membrane.

### Protein identification by mass spectrometry

Briefly, human brain extract was separated on a 10% SDS-PAGE. After Coomassie staining, the band analogous to section 10 was cut and analyzed by peptide mass fingerprinting. Bands were excised from gels using ProPic Investigator (Genomic Solutions, Ann Arbor, MI, USA) and collected in 96-well plate. Destaining, reduction, alkylation, trypsin digestion of the proteins followed by peptide extraction were carried out with the Progest Investigator (Genomic Solutions, Ann Arbor, MI, USA). After desalting step (C18-μZipTip, Millipore) peptides were eluted directly using the ProMS Investigator, (Genomic Solutions, Ann Arbor, MI,USA) onto a 96-well stainless steel MALDI target plate (Applied Biosystems/MDS SCIEX, Framingham, MA, USA) with 0.5 µl of CHCA matrix (5 mg/ml in 70% ACN/30% H2O/0.1% TFA) [38]. *MS and MS/MS analysis*: Raw data for protein identification were obtained on the 4800 Proteomics Analyzer (Applied Biosystems/MDS SCIEX, Framingham, MA, USA) and analyzed by GPS Explorer 2.0 software (Applied Biosystems/MDS SCIEX, Framingham, MA, USA). For positive-ion reflector mode spectra 3000 laser shots were averaged. For MS calibration, autolysis peaks of trypsin ([M+H]+  = 842.5100 and 2211.1046) were used as internal calibrates. Monoisotopic peak masses were automatically determined within the mass range 800–4000 Da with a signal to noise ratio minimum set to 30. Up to twelve of the most intense ion signals were selected as precursors for MS/MS acquisition excluding common trypsin autolysis peaks and matrix ion signals. In MS/MS positive ion mode, 4000 spectra were averaged, collision energy was 2 kV, collision gas was air and default calibration was set using the Glu1-Fibrino-peptide B ([M+H]+  = 1570.6696) spotted onto fourteen positions of the MALDI target. Combined PMF and MS/MS queries were performed using the MASCOT search engine 2.1 (Matrix Science Ltd., UK) embedded into GPS-Explorer Software 3.5 (Applied Biosystems/MDS SCIEX, Framingham, MA, USA) on the NCBInr database (downloaded 2008 10 22, 7135729 sequences;2462332163 residues) with the following parameter settings: species: homo sapiens, mono charged peptides, 50 ppm peptide mass accuracy, trypsin cleavage, one missed cleavage allowed, carbamidomethylation set as fixed modification, oxidation of methionines was allowed as variable modification, MS/MS fragment tolerance was set to 0.3 Da. Protein hits with MASCOT Protein score ≥65 and a GPS Explorer Protein confidence index ≥95% were used for further manual validation.

### Antibodies absorption experiments

The 96 wells flat-bottomed microtiter plates (NUNC, Denmark) were coated with 5 µg/ml of beta tubulin (TBB), beta tubulin III (TBB3) and Glial Fibrillary Acidic Protein (GFAP) and left for adsorption at 4°C overnight. The assay was performed on these plates after blocking with PBS-1% Gelatin and washing with PBS-0.1% Tween 20. Briefly, coated wells were incubated with serum samples (Pool of CM and EC sera) or monoclonal antibody (mAb) anti-TBB3 as positive control diluted at 1∶100 and 1∶500 respectively in PBS-1% Gelatin, 0.1% Tween 20 for 1 hour at 37°C. Following incubation, wells were washed 5 times with PBS-0.1% Tween 20 and the plates were then incubated with peroxydase-labeled anti-human IgG (1∶10000 in PBS-1%Gelatine, 0.1% Tween 20) and anti-mouse IgG at 37°C for 1 h respectively. Each supernatant was consecutively submitted 40 times to the same treatment. The process of revelation was identical to the one for total IgG and IgM.

Following depletion assays, all serum samples including mAb TBB3 and control non-depleted sera and mAb TBB3 were blotted on membranes containing human brain antigens separated on 10% SDS-PAGE. The immunoglobulin reactivities were detected by incubation with a γ chain-specific secondary rabbit anti-human IgG and rabbit anti-mouse IgG coupled to alkaline phosphatase (Sigma-Aldrich, France). Revelation was done by using BCIP/NBT and then dried membranes were scanned with a high resolution scanner (600 DPI).

### Statistical analysis

Immunoblot data were analyzed by multivariate statistical methods, using IGOR software (Wavemetrics, Lake Oswego, OR), including specially written software packages. The standard migration scale was divided into sections around individual peaks of immunoreactivity. Section-wise absorbance values were subjected to principal component analysis (PCA), based on covariance calculation. For quantitative comparisons between groups, we used either Mann-Whitney (between two groups) or Kruskal-Wallis tests (>2 groups). Qualitative association was tested by Pearson's χ^2^ test. The association between continuous quantitative parameters was assessed by linear regression, with the exception of correlations between two different types of parameters such as reactivity and cytokine profiles, which were tested by Spearman's rank correlation. The p values<0.05 were considered significant.

Correspondence analysis (pcc) was performed after singular value decomposition (SVD) of the different distance matrices. Inertia of the dimensions are expressed as percentages. The results of the decomposition of the principal dimensions are expressed as relative contributions of each variable, or the relative contribution of a arithmetic mean of a group of variables, to the principal dimension under study. Two-way complete-linkage hierarchical clustering (HC) based on Euclidean distances was used to analyze the relationship between the clinical groups and section cross-reactivity of the antibody preparations. SDV, pcc, PCA, and HC, as well as plotting of the results were performed using proprietary software.

## Results

### Demographic profiles of malaria patient groups

Ninety eight *P. falciparum* infected individuals were included in the present study. Selection according to the clinical variants shown that among, 16 patients corresponded to the mild malaria (MM), 10 to the severe non-cerebral malaria (SM) and 42 to CM. In controls, 5 individuals were classified in the ex-CM, 11 in the non-endemic controls (NEC) and 14 in the endemic controls (EC) groups. The demographic characteristics of each group are shown in the Table 1. Males and females were 65 and 33 respectively; a median age was 30 years (range 7–70). The NEC individuals were from a non-endemic area of *P. falciparum* and are individuals from laboratory staff that did not contact disease during at least the 5 preceding years. No parasitemia was detected in the EC, NEC or in the ex-CM groups at the time of inclusion in the study. There were no mixed infections. The median level of *P. falciparum* in the blood of patients from the infected groups (MM, SM and CM) was 1.5, 1 and 2 respectively but no statistical difference was observed between infected groups.

**Table 1:** Demographic profiles of the *P. falciparum* malaria patient groups.

Groups	Patients no. (%)	Median age (range)	Median Parasitemia % (range)	Sex (M/F)
NEC	11 (11,2%)	35 (25–63)	0	10/1
EC	14 (14,3%)	26 (23–37)	0	13/1
MM	16 (16,3%)	30 (15–45)	1,5 (0,1 – 4,25)	9/7
SM	10 (10,2%)	30 (8–65)	1 (0,1 – 15)	7/3
CM	42 (42,9%)	36 (9–70)	2 (0,25 – 60)	22/20
ex-CM	5 (5,1%)	24 (7–60)	0	5/0
Total	98 (100%)	30 (7–70)	0,5 (0,1 – 60)	65/33

NEC- non endemic control, EC- endemic control, MM- mild malaria, SM- severe non-cerebral malaria, CM- cerebral malaria, ex-CM- Ex-cerebral malaria.

### Total and specific IgM and IgG responses to *P. falciparum* and brain antigens according to clinical groups

**Figure 1:**
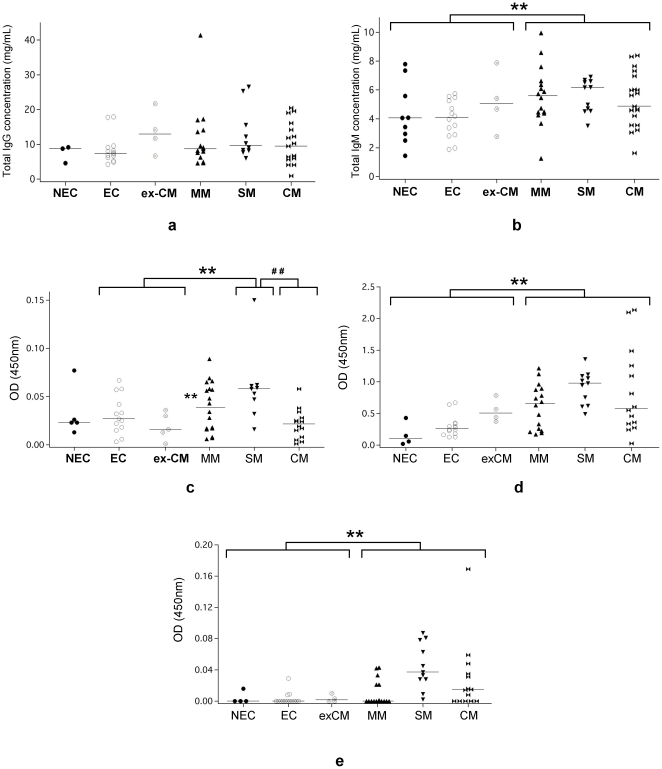
Total, brain-, and *P. falciparum*–specific IgG and IgM responses in different groups. Distribution of total levels of IgG (a) and IgM (b) in the different group of patients determined by Sandwich ELISA (** p = 0.003). Median level is indicated. Rate (optical density) of specific IgG against *P. falciparum* FAN5HS erythrocytic stage extract quantified by direct ELISA (** p = 0.008) (## p = 0.001) (c). Rate (optical density) of IgG (** p<0.001) (d) and IgM (** p = 0.002) (e) recognizing the human brain extract quantified by direct ELISA.

We assessed the levels of total IgG and IgM in sera of the different groups of patient by ELISA. Interestingly, EC, NEC and ex-CM groups exhibit similar levels of total IgM and IgG. Thus, they were considered as a unique control group of non infected patients. Median levels of total IgM in MM, SM and CM patients were significantly higher than in controls (p = 0.018, 0.02 and 0.04 respectively). It is noteworthy that no significant difference was observed between infected and control groups for total IgG levels (Figure 1A and 1B). Then, we measured the concentrations of specific IgG and IgM to *P. falciparum (FAN5HS)*. A slight but non-significant increase in the rate of specific IgG and IgM to parasite was observed in infected compared to non-infected groups. However, in CM group of patients, we observed a significant decrease of specific IgG to *P. falciparum* when compared to SM (p = 0.001) (Figure 1C).

Furthermore, when assessed the levels of IgG and IgM recognizing brain proteins in the different group of patients and controls, we found significant higher levels of antibody against brain proteins in infected groups than in the control (p<0.001 and p = 0.002 respectively) albeit their rates were significantly lower in the CM patients (Figure 1D and 1E).

Taken together, these data suggest that the efficiency to produce specific antibody response to either parasite or brain antigens is diminished in the CM patients group. Also, no significant correlations were observed between age, sex or parasitemia and the rate of total and specific IgG or IgM to *P. falciparum* and brain antigens and no relationship with disease severity and level of total or specific IgG or IgM to brain or to *P. falciparum* antigens.

**Figure 2:**
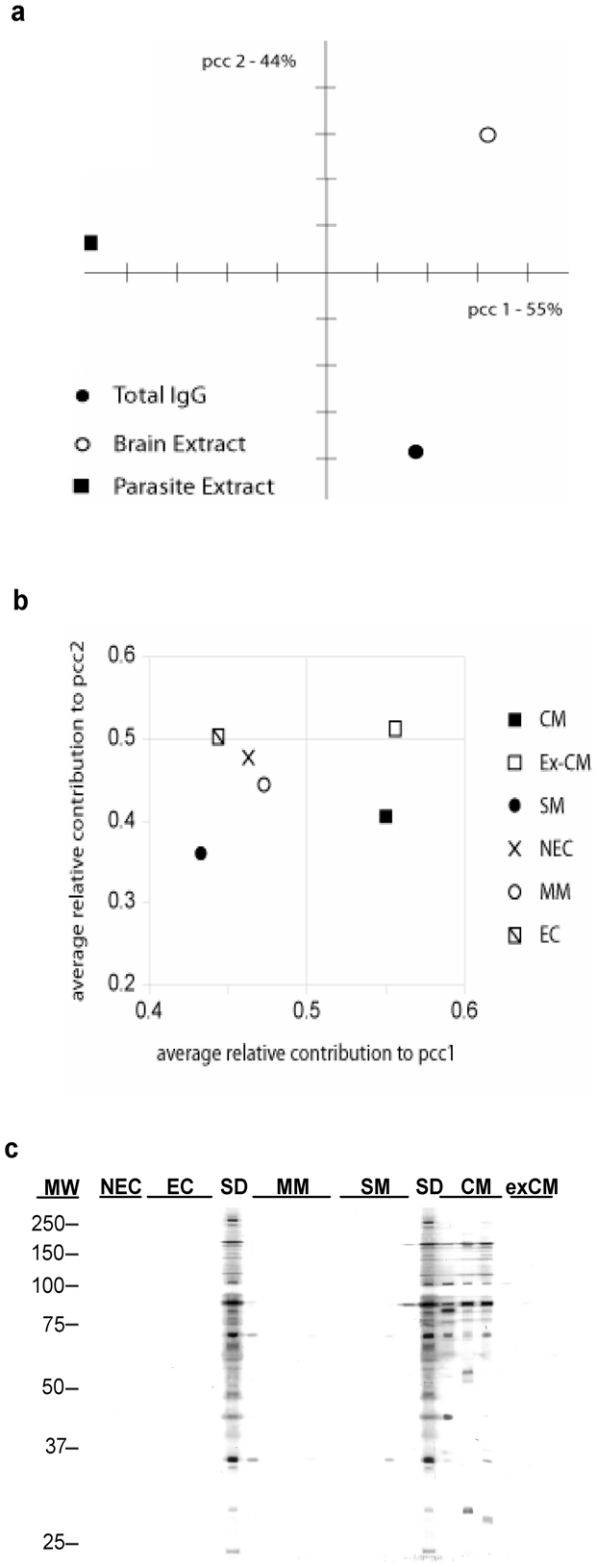
Correlation between total, brain-, and parasite-specific IgG responses. (A) Correlation-based principal component analysis of the levels of IgG responses to brain and *P. falciparum* FAN5HS antigens and total IgG determined by ELISA from all patients. (B) Relative average contributions of the different patient groups to the two principal components shown in (A). (C). Example of immunoblot showing the reactivity of IgG from patients sera from different groups with *P. falciparum* FAN5HS erythrocytic stage antigens.

We next used correspondence analysis to examine the relationship between the three types of specific antibody responses (total, parasite and brain) and clinical outcome. Correspondance analysis of the specific antibody responses measured in all patients reveals that the first principal component (pcc1, 55% inertia) separates the response to the parasite extract from the other two, and only the second principal component (pcc2, the remaining inertia) places the antibody response to the parasite roughly equidistant to both the brain and total IgG responses (Figure 2A). This result indicates that the production of antibodies against brain antigens is independent of this specific to the parasite. Decomposition of both dimensions and arithmetic averaging over patient groups reveals that pcc1 represents IgG response in CM and ex-CM, whereas pcc2 is in majority defined through response in ex-CM and EC groups (Figure 2B). Specially, the decomposition of pcc1 confirms that CM patients seem to develop lower measurable levels of antibody to parasite antigens, but broader in term of specificity as exemplified in the Figure 2C, than both SM and MM patients. Some of these *P. falciparum* specific antibodies are still detectable in ex-CM patients.

### Analysis of the serum IgG autoantibody repertoire expressed against brain antigens in patients with distinct clinical forms of *P. falciparum* malaria

**Figure 3:**
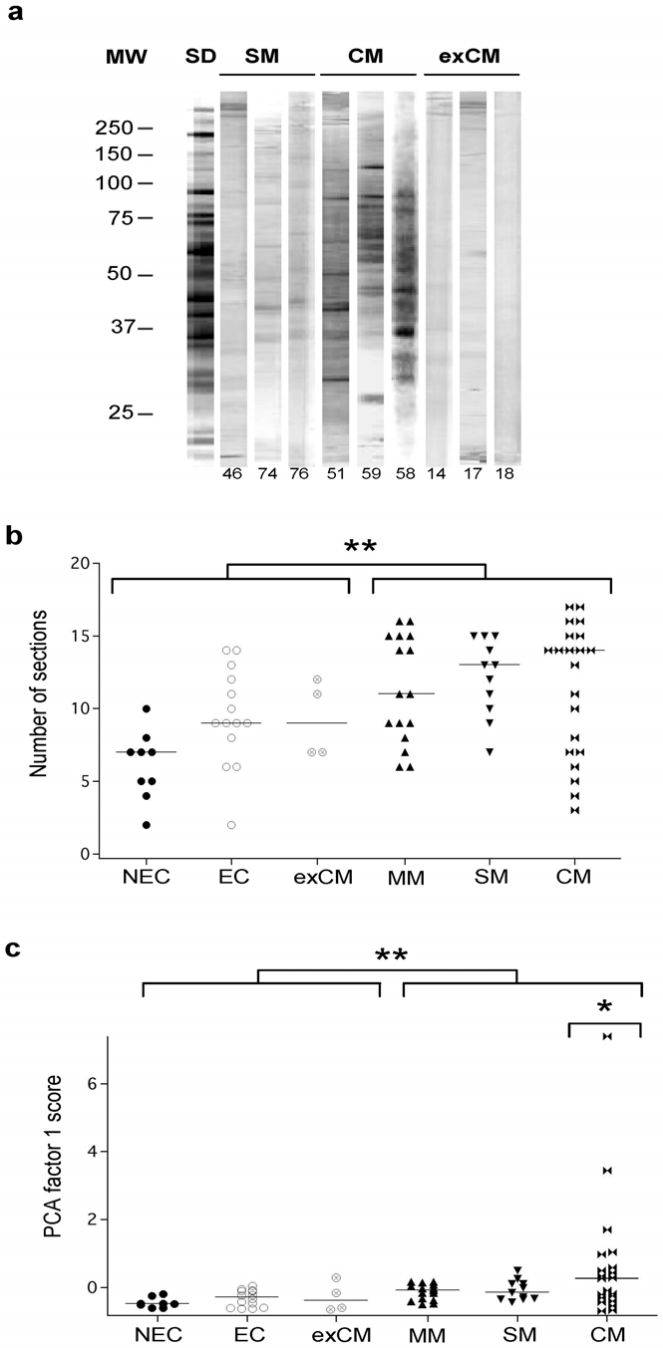
Profiles of IgG reactivities to brain antigens of the different *P. falciparum* infected groups. (A) Example of IgG reactivity from SM, CM, or ex-CM patients sera showing the increase with disease severity and the number of brain antigens (section) reacting with patient sera. (B) Median number of sections recognized by each patient from the different groups (** p<0.001). (C). PCA factor 1 score from unadjusted IgG reactiviy profiles. Groupwise distribution of PCA factor 1 scores. PCA1 score were significantly higher in infected than control groups (** p<0.001) and in CM than other groups (* p = 0.01).

We first analyzed the reactivity patterns of IgG from the different groups of malaria patients to brain protein using PANAMA-BLOTs as previously described [23]. Reactivity against brain antigens expressed by Indian patients and by the standard consisting of a pool of serum from Gabonese CM patients are shown in Figure 3A. We found a high correlation between disease severity and an increased diversity of the repertoire of antigens recognized by circulating antibodies in *P. falciparum* patients. This was principally observed in CM patients. Those patients recognized more protein sections than the other groups of individuals tested. Healthy individuals completely lack reactivity against the brain extract. It is interesting to note that the link between CM pathology and the increase of IgG reactivity to brain antigens is reinforced by the low number of sections observed in the ex-CM patients (Figure 3B). These data are in agreement with our earliest observation in children from a hyperendemic area of Gabon [23]. Optical density analysis of the profiles of reactivity on the immunoblots allow defining peaks of density which corresponds to a section of brain protein recognized by IgG from a pool of sera of Gabonese CM children constituting our standard used for adjustment [23]. Profiles of antibody reactivities were separated into 18 sections as shown in the Figure S1. Next, profiles of reactivity from each patient group were compared by principal component analysis (PCA). In PCA, the components are identified in decreasing order of importance. Thus, by definition, the first two components identified account for a large proportion of total reactivity. Factor 1 scores mostly reflected the recognition of one particular section and significantly higher in CM patients than the other groups with brain extract (p = 0.01) (Figure 3C). These results thus demonstrate a production of autoantibody to brain proteins in CM patients.

**Figure 4:**
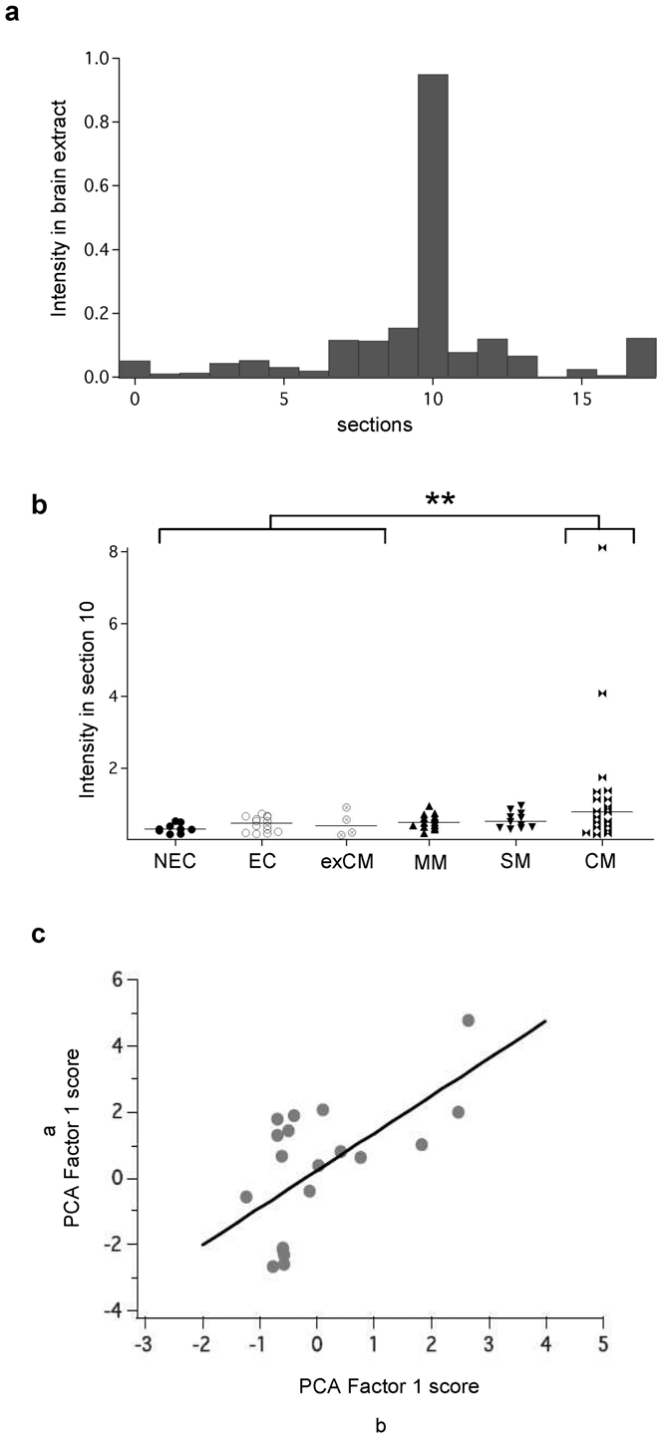
Reactivity to brain antigens of the different malaria patients group. (A) Distribution of mean intensity reactivities of IgG from CM patients with the different sections in brain extract. The section 10 is the most recognized among 18 sections. (B) IgG reactivity with section 10 is significantly higher in CM group than other groups (** p = 0.006). (C). Correlation of IgG reactvity with two different brain extracts. PCA factor 1 (α) correspond to the IgG reactivity from CM patients with Cuban healthy brain extracts (β) represent the reactivity of a commercial protein medley sample. Correlation coefficient: R = 0.6237, Regression: p = 0.003.

In CM group, section 10 which corresponds to proteins of approximately 50 kDa, had maximum impact; more than 90% of total reactivity corresponds to PCA factor 1 (Figure 4A). In addition, the mean intensity of serum IgG reactivity to brain against antigens of section 10 was significantly higher in CM patients than control groups (p = 0.006) (Figure 4B). In order to exclude alloreactivities, we used another source of brain antigens, the Medley brain protein extract. Results obtained were similar than those found with the previous extract. A high significant correlation between reactivities expressed by the different group of patients with the two brain antigen sources was calculated (r = 0.623, p = 0.003). This observation thus suggests that the IgG reactivity against brain proteins observed during malaria is irrespective of the brain donor (Figure 4C).

We also compared the average ability of sera from Indian and from our previous published Gabonese CM patients data to react with the same brain antigen extracts [23]. Interestingly, their profiles of reactivity to the brain extract overlaid suggesting that these different groups of patients originating from India or Gabon recognize the same spectrum of brain proteins. Nevertheless, the Gabonese CM group show a dominant reactivity with proteins of the section 0 while the Indian CM patients are distinguished by their predominant reactivity with proteins of the section 10 even if they also recognize section 0 (Figure S2).

**Figure 5:**
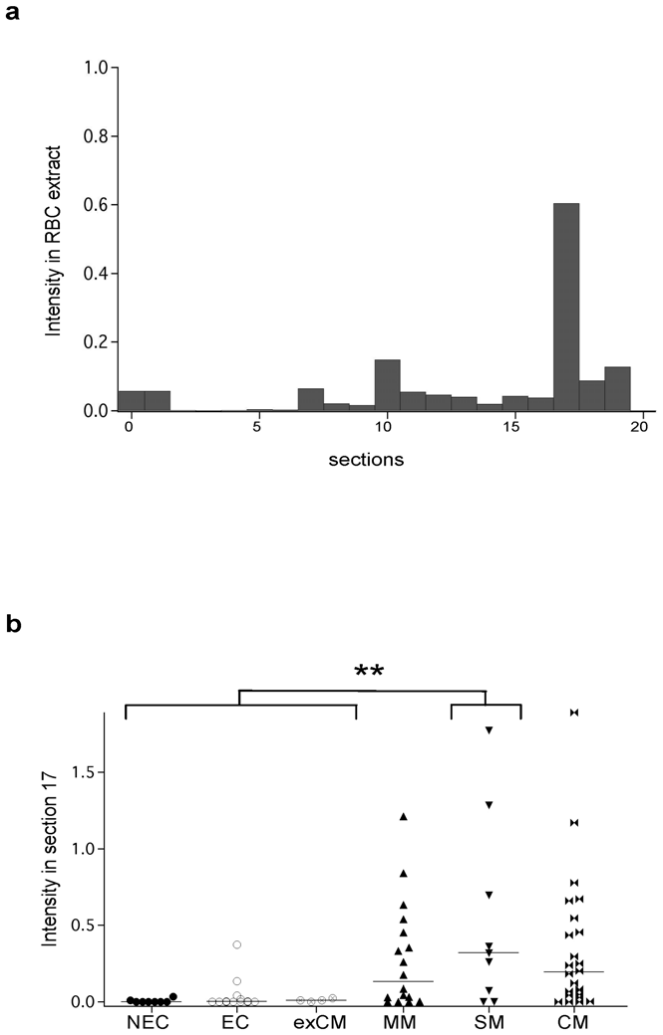
Reactivity to non-infected red blood cell antigens of different groups of patients. (A) Distribution of the mean intensity of IgG reactivity to RBC protein extract separated into sections (B) mean intensity of IgG reactivity to section 17 is significantly higher in SM than in the other groups (** p = 0.002).

To assess if the reactivity with section 10 in CM patients is specific to the brain tissue, we analysed the patterns of reactivity of same sera with RBC protein extract. An example of reactivity of patient serum IgG against RBC protein extract is shown in the Figure S3A. Statistical analysis reveal high differences between infected patients and controls (p<0.001) (Figure S3B). However, no statistical differences were observed when comparing groups of infected patients (MM, SM and CM). Profiles of reactivity of patient plasma samples with RBC proteins were separated into 20 sections according to the standard used for brain extract. However, PCA analyses allow us to identify the section 17 contributing to the difference between groups but not the section 10 identified in brain extract (Figure 5A). Moreover, the mean intensity of the IgG reactivity to section 17 was significantly higher in SM than control groups (p = 0.002) (Figure 5B). These results suggest that the IgG reactivity to antigens in brain section 10 could be a signature of CM patients. It is noteworthy that no significant correlation was observed between the reactivity profile to brain antigens represented by PCA factor 1 scores and age, parasitemia or sex. Besides, levels of total, brain or parasite specific IgG do not correlate with brain autoreactivity profiles.

**Figure 6:**
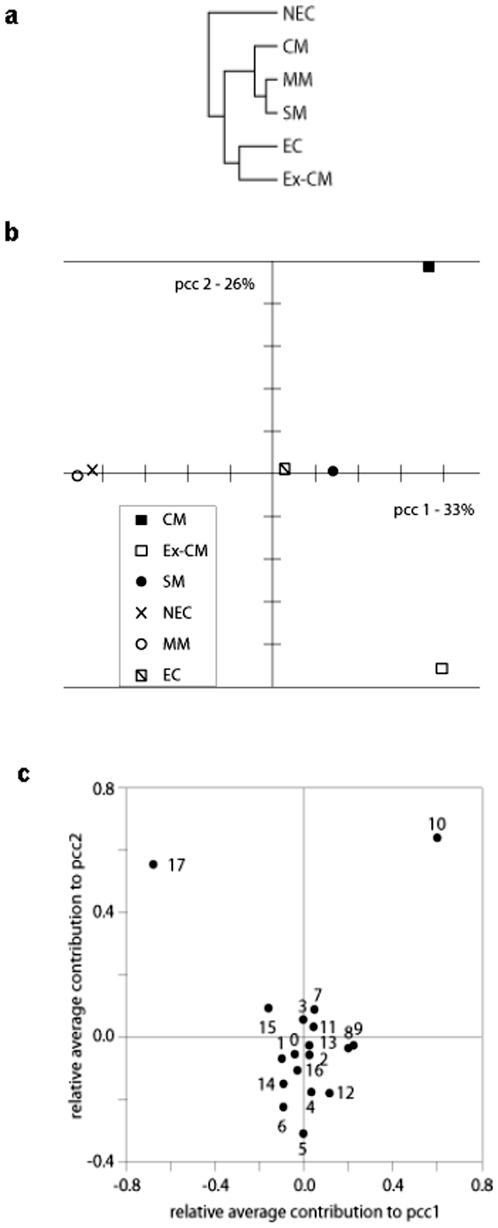
Reactivity to section 10 distinguishes cerebral malaria. (A) Hierarchical clustering of malaria patient groups according to their reactivity with brain proteins analyzed by PANAMA blots. (B) Correlation analysis of malaria patient group IgG reactivity. (C) Decomposition of correlation analysis.

Importantly, when arithmetically averaged over the patient groups, the reactivity of patient sera to the different sections on the blots can be used to classify the different patient groups using two-fold complete-linkage hierarchical clustering (Figure 6A). As would be expected, *P. falciparum* infected patients and the control groups form distinct clusters. Interestingly, among malaria affected groups, SM and MM are more closely related amongst each other than with CM. Also, it is worthy to note that the close relationship between EC and ex-CM sets them apart from the NEC group, indicating a possible contamination of the EC group with undetected ex-malaria cases. Correspondence analysis of IgG reactivity to brain antigens of the patient groups according to their average response to the eighteen different sections (Figure 6B) reveals that the second resulting principal component (representing 26% of total inertia) is almost solely responsible for separating the ex-CM and CM patient groups from the control and other *P. falciparum* infected groups. Decomposition of the first two principal components (Figure 6C) demonstrates that sections 10 and 17 account for the majority of pcc2 whereas section 17 has the least and section 10 the principal contribution to pcc1. Taken together, this dimensionality reduction analysis firmly establishes the predominance of section 10 and 17 in distinguishing CM from ex-CM and of section 10 in defining CM.

Note that the contributions of the individual sections are expressed as relative measures with the barycentre at (0,0). No significant contribution of any other section is observed. Therefore, IgG reactivity to antigens in section 10 and 17 could be biomarkers of ex-CM cases whereas IgG reactivity to section 10 could be used as a disease-marker for CM.

### TBB3 is a major discriminant antigen recognized by serum IgG of CM patients

**Figure 7:**
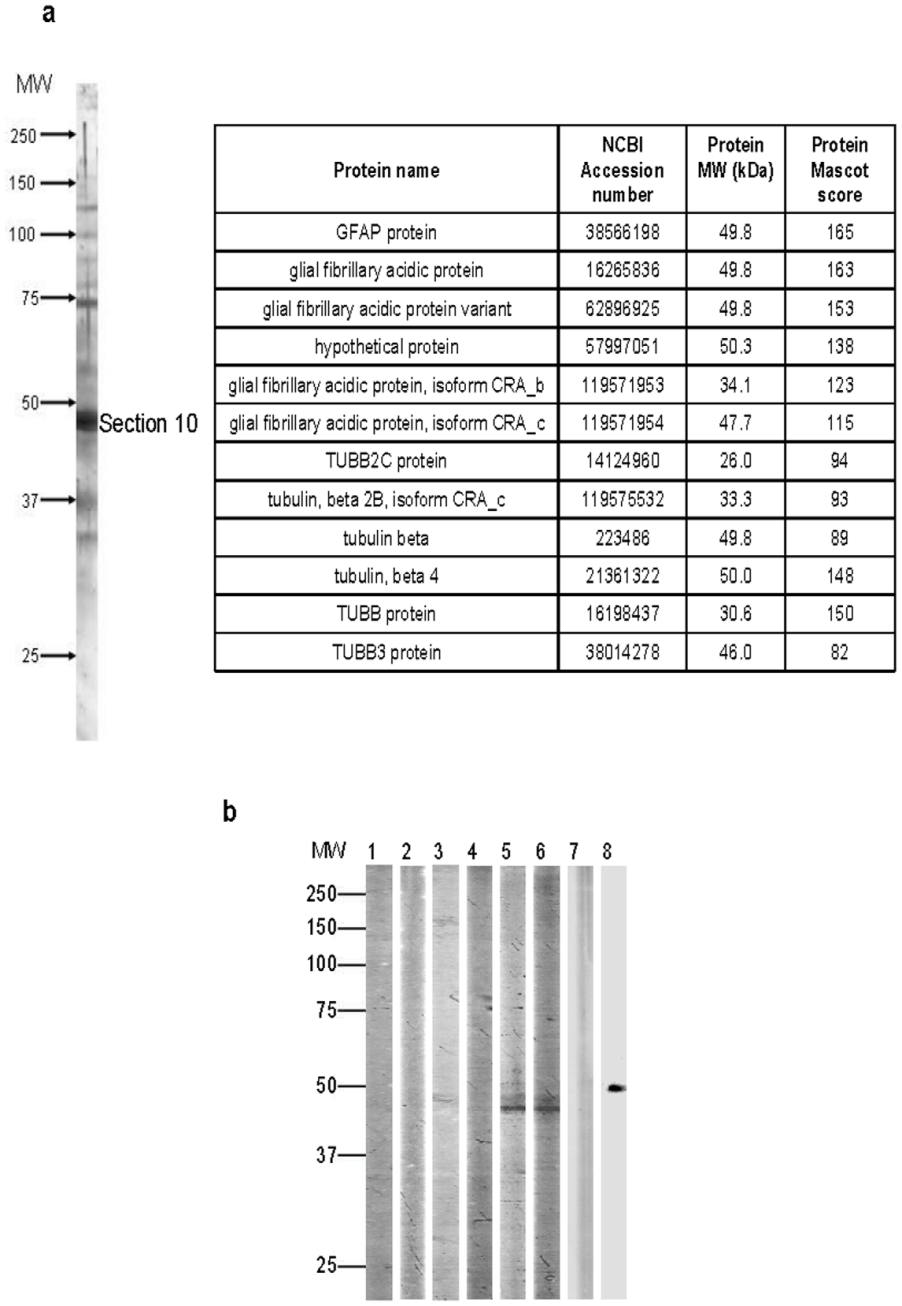
Identification and characterization of proteins contain in the section 10. (A) Protein identification by mass spectrometry. Twelve human proteins identified from the section 10. Identification of proteins was carried out as described (see material and methods). In this case, Mascot protein scores greater than 65 are significant (p<0.05). (B). Characterization of section 10 protein candidate by antibody depletion. Immunoprinting with sera depleted (d) or not depleted (nd) with TBB, TBB3, and GFAP proteins. No signal was detected at 46 kDa in CM sera depleted with TBB and TBB3 proteins in lane 3 and 4 respectively. MW, Molecular weight marker; 1, EC sera (nd); 2, EC sera (d); 3, CM sera (d) with TBB; 4, CM sera (d) with TBB3; 5, CM sera (d) with GFAP; 6, CM sera (nd); 7, TBB3 mAb (d) with TBB3; 8, TBB3 mAb (nd).

Furthermore, candidate proteins in section 10 were identified using mass spectrometry. In three independent experiments based on matching of peptide mass, the family of Beta Tubulin (TBB), in particular TBB3 specifically expressed in the brain and Glial Fibrillary Acidic Protein (GFAP) were identified as discriminant antigens using the Swiss-Prot database. However, due to the structural homologies between the several tubulin isotypes, it was not possible to distinguish by mass spectrometry if one or several isoforms of TBB were present in this section (Figure 7A). Therefore, additional analyses were performed to specifically analyze the involved tubulin isotypes. To validate the mass spectrometry results we further depleted sera samples from CM and EC with TBB3, TBB or GFAP proteins. After 40 rounds of depletion of 1 hour each, levels of antibodies recognizing TBB3, TBB or GFAP were quantified in the depleted samples by ELISA. A decrease of the level of specific antibodies with round number to the respective proteins was observed except for GFAP demonstrating that we can exclude GFAP as a candidate (data not shown). It is noteworthy that 40 rounds of depletion were necessary to remove TBB or TBB3 specific antibodies in CM sera whereas only 7 rounds were sufficient for EC samples. In addition, the recognition of section 10 by depleted serum samples was analysed by Western blot (Figure 7B). No signal at 46 kDa was detected in CM samples depleted with TBB3 or TBB after 40 rounds whereas a signal was still detectable when the membrane was blotted with GFAP depleted sera. In addition, no signal was seen in depleted EC sera and TBB3 monoclonal antibody (Figure 7B). Taken altogether these results indicate that TBB3 is a discriminant autoantigen targeted by IgG in CM patients.

### Relationship between IgG reactivity to brain proteins and cytokine activity patterns in *P. falciparum* malaria

**Figure 8:**
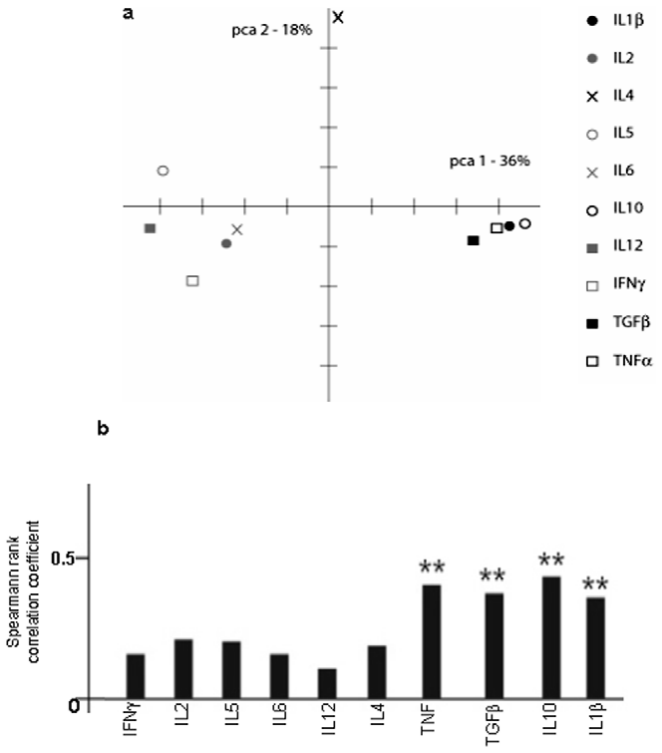
Correlation of total IgG reactivity against brain with cytokine profiles. (A) Principal component analysis (PCA) of the Spearman rank correlations between cytokine levels and IgG reactivity towards the eighteen sections of the Panama blots for each subject in our patient cohorts. We considered the entire panel of cytokines used in our previous study (32). (B) Distribution of Spearman rank correlations of PCA factor 1 scores between IgG self-reactivity in CM patients to brains proteins and the levels of cytokines from cluster 1 (IL2, IL5, IL6, IL12, and IFN-γ) and 2 (IL1β, IL10, TNFα, and TGFβ) quantified by ELISA.

Cytokines are thought to play an important role in malaria pathogenesis, particularly in CM (11). The relationship between the clinical severity of malaria and the complex pro- and anti-inflammatory cytokine network has been addressed in the same cohorts of *P. falciparum* infected patients and the results were previously published [32]. Among the 12 cytokines quantified, a coupled 2-way clustering and discriminant analyses allowed identification of a cluster cluster-II cytokines (IL1β, IL10, TNFα and TGFβ) that displays significantly increased (p<10^−6^) activity in the CM group compared to other groups, and which can be used to differentiate between different clinical forms of malaria and control groups [32]. As we show here, similarly TBB3 auto-antigen presence is a marker for CM and immune-reactivity of subjects can be used to classify different clinical forms of malaria as well as control groups. We therefore wanted to know whether or not both markers truly correlate and are surrogates. To this end we calculated Spearman rank correlations between cytokine reactivity and immune-activity towards the 18 sections on the blots for each subject in our patient cohorts. We considered the entire panel of cytokines used in our previous study [32]. When the resulting Spearman rank correlation distance matrix is singular value decomposed and analyzed for its principal covariance-based components, the cluster-II cytokine TGFβ, TNFα, IL10, and IL1β, also form a distinct cluster (Figure 8A). Therefore, the correlation between cluster-II cytokine levels and total immuno-reactivity to the different sections on the immune-blots is sufficiently strong to allow distinction. The Spearman rank correlation for the cluster-II cytokine activities and the immune-reactivity to section 10 thereby is highly significant (Figure 8B). In conclusion, IgG reactivity to TBB3 in central Indian malaria patients is statistically significantly correlated with the cluster-II cytokine levels which we had previously shown to be a marker for cerebral malaria in the same population.

## Discussion

Recently, we have shown autoantibodies to α-II spectrin of the brain in the serum of Gabonese *P. falciparum* infected children with CM [23]. Nevertheless, the exact nature of this response remains elusive. Considering the multifactorial character of malaria, the purpose of the present study was to validate these findings by generalizing our analysis to an Indian population with different genetic background, endemic and environmental status. The presence of autoantibodies in malaria patients has long been recognized but their role in the pathophysiology of CM is very little explored and not defined [22], [23], [28].

We have used a global approach aiming not only at studying the individual components involved, but also the complex interactions between these components, in order to elucidate the global nature of autoantibody response to brain antigens produced in patients with different clinical spectra of malaria. The population studied was from Gondia in the central India where *P. falciparum* malaria is epidemic [39]. In the groups of patients studied, the most severe form of the disease is developed for the greater part in 30-year-old adults on average. This could mainly due to the fact that the majority of these patients were seasonal workers staying in the region of Gondia only during the periods of harvest. Gondia is a zone where the spread of *P. falciparum* is rather recent. Most of the patients studied developed their first *P. falciparum* malaria episode and do not present a mixed infection. In our population of study and, in agreement with previous reports, the parasitemia rate alone was not enough to evaluate the severity of the disease since it was equivalent in CM than in SM and MM groups [40].

Polyclonal B cell stimulation through parasite mitogens coupled with the secretion of parasite specific antibodies can explain the higher amount of total antibodies observed in infected compared to control groups [25], [26], [28]. Similar observations have been made when analysing the autoantibody response to brain antigens among the various groups of patients. The group of CM presents the lowest specific IgG and IgM reactivities to brain proteins while those are increased when infected versus non infected group of patients are compared. As demonstrated and confirmed by the correspondence analysis, the antibody-mediated immune response to brain proteins detected in *P. falciparum* infected patients seems to be mainly due to a selective and inducible process during the infection. The disappearance of these autoantibodies in the ex-CM group reinforces this hypothesis. As revealed by pcc1 factor (Figure 1), the antibody response to brain antigens is largely independent of the parasite specific response. In addition, there is no relationship with disease severity and total antibody levels, neither with specific IgG or IgM to brain or to *P. falciparum* antigens. Overall, these observations suggest that the spontaneous autoantibody production against the brain during malaria carries the hallmark of a typical immune response induced by parasite infection.

Interestingly, reactivity to all 18 sections of the brain extract with circulating IgG from the different individuals of the cohorts is sufficient to comprehensibly cluster the different patient groups as demonstrated by the hierarchical clustering analysis. Decomposition analysis reveals that reactivity with section 10 in CM patients is mainly responsible for this classification capacity. Brain specificity of the IgG response to section 10 in CM patients has been demonstrated by the lack of reactivity to the same section when normal RBC protein extract has been used as antigen. Thus, IgG reactivities against human brain and RBC extracts strongly suggest that the development of autoimmune antibodies is more noticeable in patients who develop CM than in the other group of malaria patients.

This study highlights the important finding of the increase of the repertoire of brain antigens recognized by IgG of Indian CM patients. These results validate and extended our previous observations in Gabonese patients [23]. They also strongly support the hypothesis that an antibody-mediated self-reactivity to brain antigens triggered during *P. falciparum* infection is associated with cerebral malaria. However, we do not know yet if this antibody response is an aggravating factor that contributes to the development of cerebral malaria or is one of the consequences of the syndrome. Nevertheless, on the contrary to the Gabonese CM patients mostly characterized by an autoantibody response directed to α-II spectrin, Indian CM patients showed strong reactivity with the human brain proteins TBB3 identified by mass spectrometry in section 10. It is noteworthy that only some Indian CM patients recognize the α-II spectrin. This observation indicates a particularity of IgG self-reactive response to brain proteins in the Indian population. The correlation of the profiles of reactivity of CM patients to two different brains extracts point out that the profile of IgG reactivity to brain cannot be explained by an alloreactive response. Opposite to the observations made in the study with *P. falciparum* infected children from Gabon, no correlation was found between the age, the sex, parasitemia and concentration of total IgG, and the IgG autoreactivity to brain antigens.

TBB3 is a cytoskeleton protein, which is abundant in the central and peripheral nervous systems (CNS and PNS) and expressed during fetal and postnatal development. In adult tissues, TBB3 is mainly expressed in the brain and PNS and used as a neuron-specific marker molecule encoded by a gene located at the long arm of chromosome 16 in man [41] thereby highlighting a possible pathogenic role between such autoimmune response and the occurrence of CM. In support of this interpretation, no significant increase in anti-tubulin antibody levels was seen in sera of patients infected with *Plasmodium vivax* or with tuberculosis [42]. However, the level of serum anti-tubulin antibodies was significantly elevated during infectious diseases such as visceral or cutaneous leishmaniasis, onchocerciasis, schistosomiasis and leprosy, but it is unknown if such autoantibodies involve a reaction against TBB3 [42].

A two-way coupled cluster analysis revealed 2 clusters of cytokines relevant to clinical subgroups of disease in the same cohorts of malaria patients studied [32]. In particular, the significant abundant level of cluster-II cytokines (TGFβ, TNFα, IL10 and IL1β) was relevant to the discrimination of CM from SM. Importantly, we have shown that cluster-II cytokine levels strongly correlate with reactivity to TBB3 in CM. The fact that we have been able to classify the different malaria and control groups based on the statistically significant IgG reactivity to TBB3 associated with cluster II cytokines despite the relatively small size of our cohort, demonstrates the prevalence of this autoantibody-mediated reactivity in CM and therefore its clinical relevance.

To summarize, the IgG response against TBB3 found in CM could be a new biomarker of CM in the Indian population. While the molecular mechanisms of antibody production to TBB3 during *P. falciparum* infection remain unknown, the study of this phenomenon potentially leads to new avenues in the understanding of malaria physiopathology. Despite these findings, a longitudinal study of malaria clinical states, in conjunction with studies of cytokine production, specific and self-reactive antibody responses and several other biological parameters on largest populations from endemic and epidemic areas of India would appreciably add to our understanding of the role of immune responses in general in disease severity associated with *P. falciparum* infection. We have here established the basis for such a deeper investigation.

## Supporting Information

Figure S1 Determination of sections. Localizations of the bands on Western blot profile of different groups obtain after the computer analysis of membrane N19 and sections are defined using the IgG reactivity of standard (pool of Gabonese CM patients). Bands are ordered from high to low molecular weight (between about 230 kDa and 20 kDa).
(1.05 MB TIF)

Figure S2 Comparison of IgG reactivities within different clinical groups with section 0. The mean intensity of IgG reactivity in different groups of patients with section 0 (* p = 0.012) (** p = 0.018).
(1.04 MB TIF)

Figure S3 Profiles of IgG reactivity in different clinical groups of patients with RBC extract. (a) A blot represents increase IgG immunoreactivity in CM patients than others (b) Groupwise distribution of PCA factor 1. The PCA1 score was significantly higher in infected than control groups (** p<0.001)
(1.17 MB TIF)
