# Supplementary figures and images for: IgG Autoantibody to Brain Beta Tubulin III Associated with Cytokine Cluster-II Discriminate Cerebral Malaria in Central India
Source: PLoS One. 2009 Dec 14;4(12):e8245. doi: 10.1371/journal.pone.0008245 (PMC2788233; doi:10.1371/journal.pone.0008245)

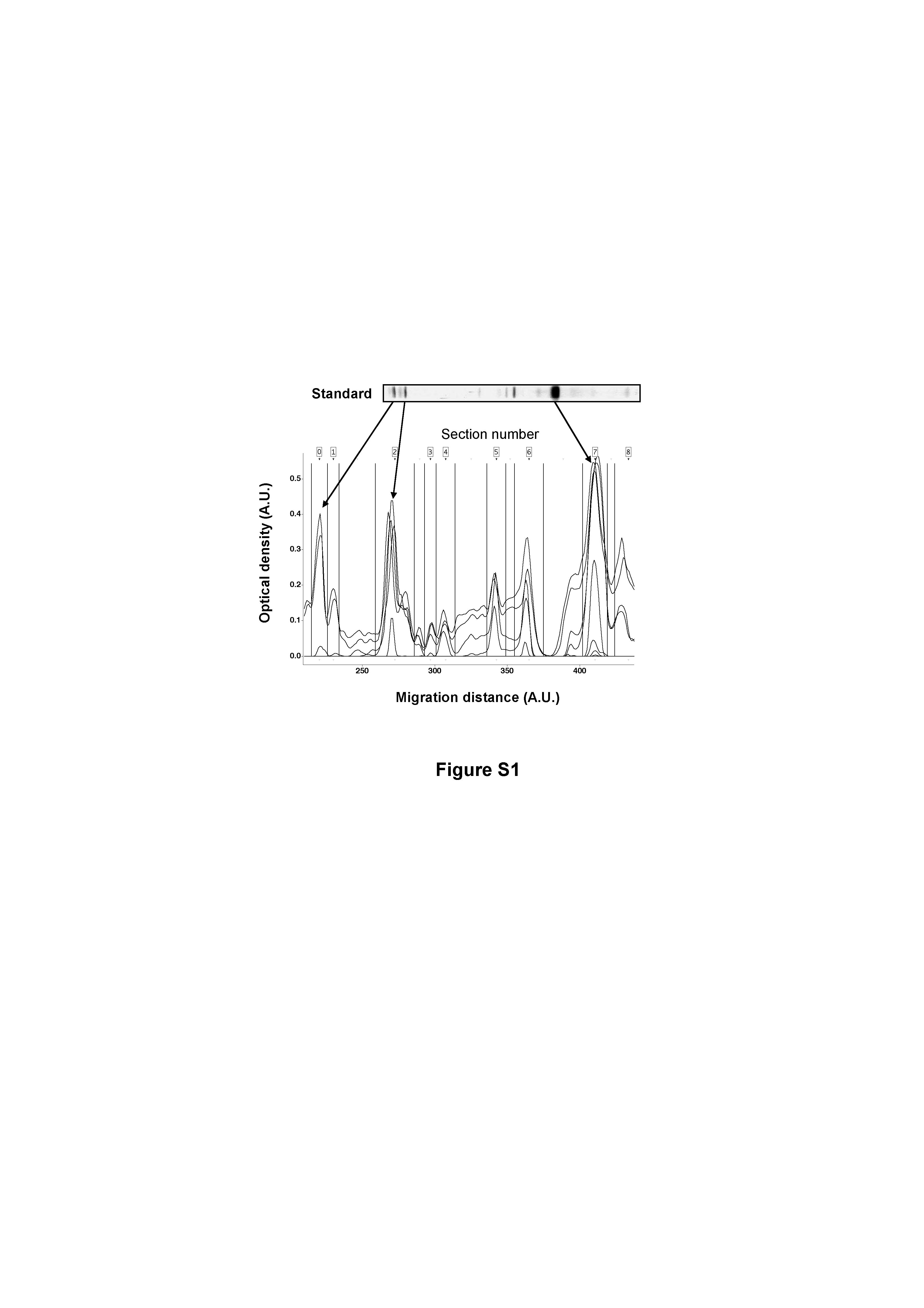

Supplement: Figure S1 — Determination of sections. Localizations of the bands on Western blot profile of different groups obtain after the computer analysis of membrane N19 and sections are defined using the IgG reactivity of standard (pool of Gabonese CM patients). Bands are ordered from high to low molecular weight (between about 230 kDa and 20 kDa). (1.05 MB TIF) [file pone.0008245.s001.tif]

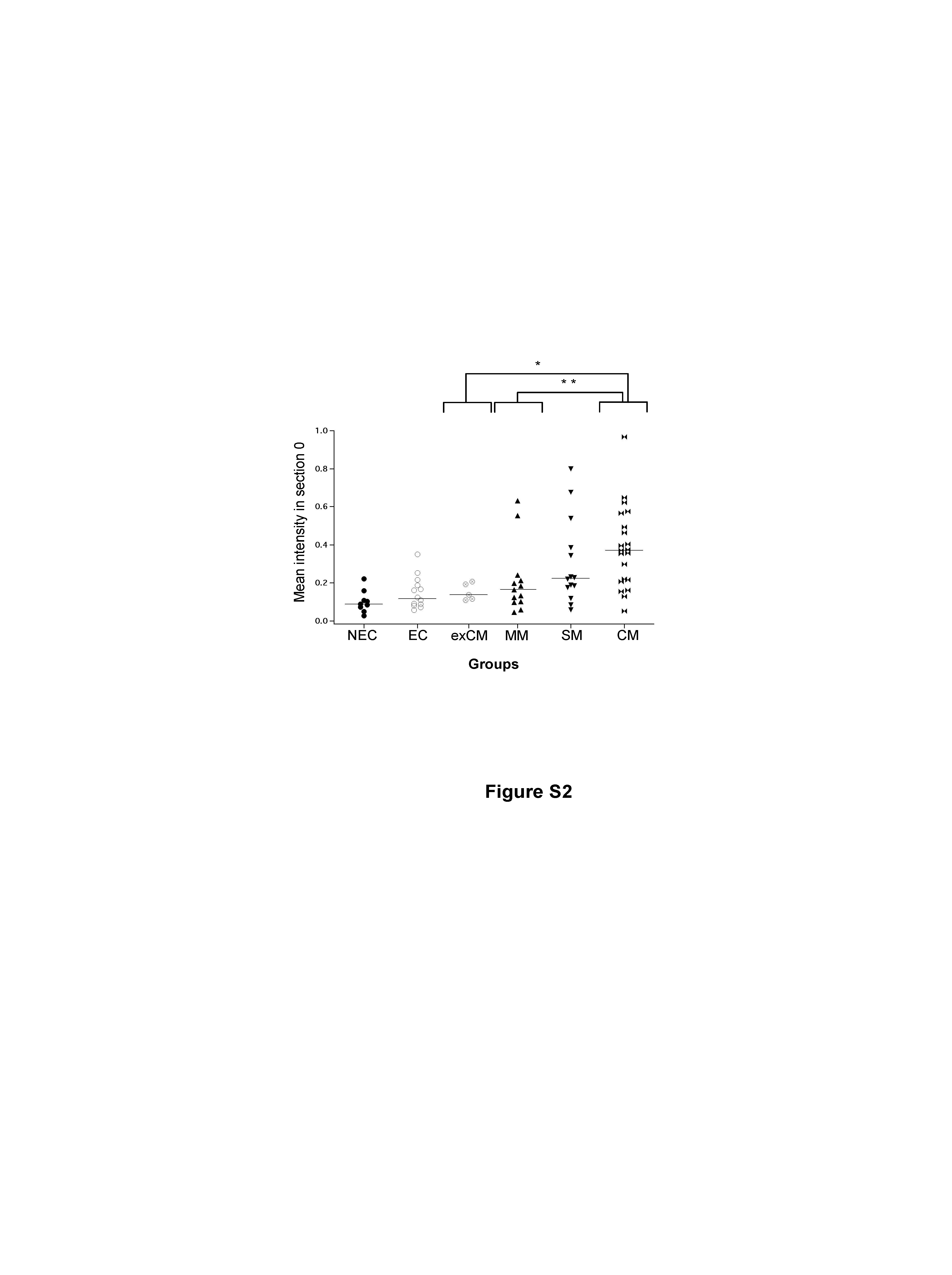

Supplement: Figure S2 — Comparison of IgG reactivities within different clinical groups with section 0. The mean intensity of IgG reactivity in different groups of patients with section 0 (* p = 0.012) (** p = 0.018). (1.04 MB TIF) [file pone.0008245.s002.tif]

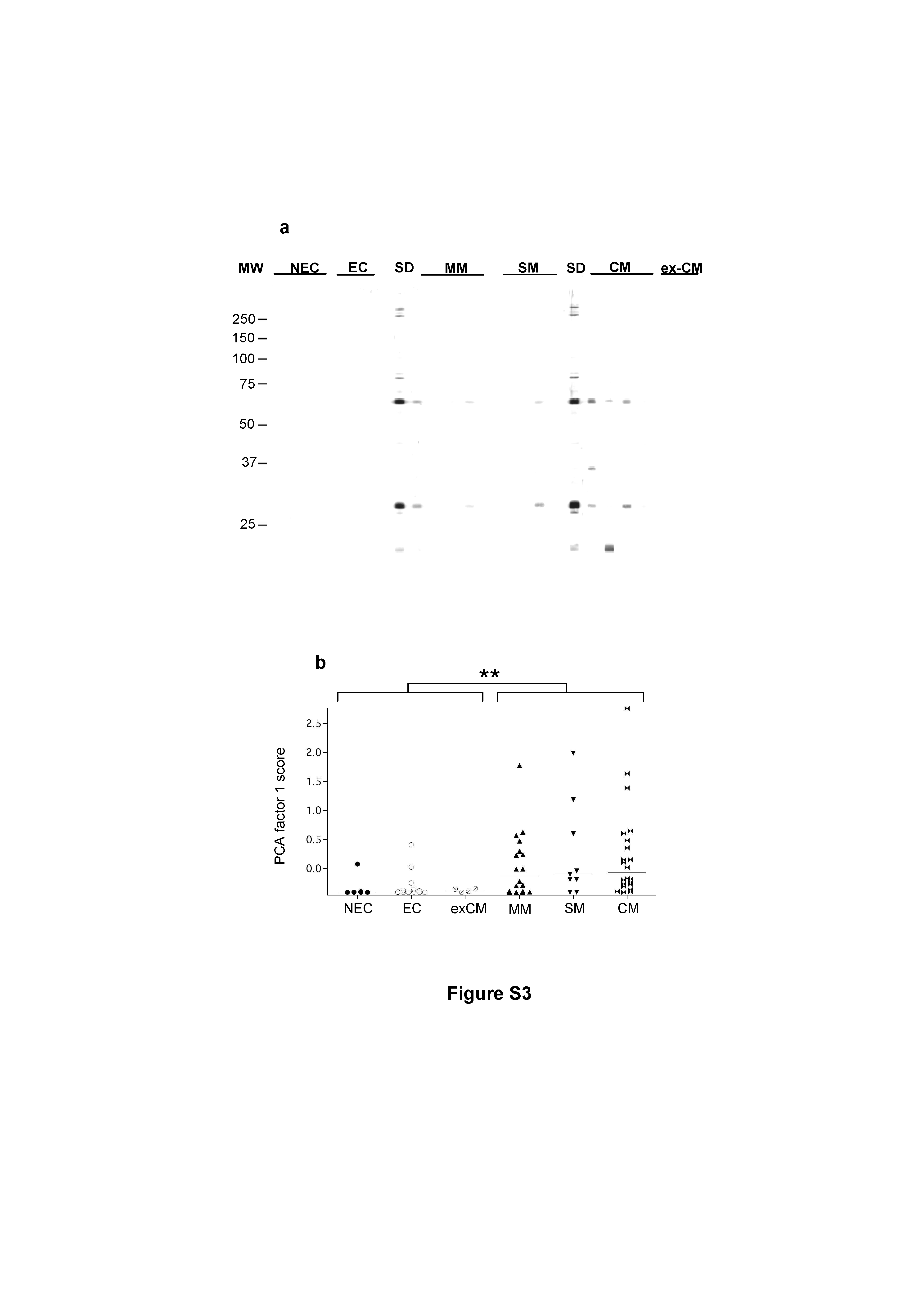

Supplement: Figure S3 — Profiles of IgG reactivity in different clinical groups of patients with RBC extract. (a) A blot represents increase IgG immunoreactivity in CM patients than others (b) Groupwise distribution of PCA factor 1. The PCA1 score was significantly higher in infected than control groups (** p<0.001) (1.17 MB TIF) [file pone.0008245.s003.tif]
